# Impact of Salt on Thermal Stability and Dose Response of the Fricke-XO-Pluronic F-127 3D Radiotherapy Dosimeter

**DOI:** 10.3390/ma15155223

**Published:** 2022-07-28

**Authors:** Michał Piotrowski, Piotr Maras, Radosław Wach, Slawomir Kadlubowski, Marek Kozicki

**Affiliations:** 1 Department of Mechanical Engineering, Informatics and Chemistry of Polymer Materials, Lodz University of Technology, 90-543 Lodz, Poland; michal.piotrowski@dokt.p.lodz.pl; 2 Department of Radiotherapy Planning, Copernicus Hospital, Pabianicka 62, 93-513 Lodz, Poland; piotr.maras@wp.pl; 3 Institute of Applied Radiation Chemistry, Chemistry Faculty, Lodz University of Technology, Wroblewskiego 15, 93-590 Lodz, Poland; radoslaw.wach@p.lodz.pl (R.W.); slawomir.kadlubowski@p.lodz.pl (S.K.)

**Keywords:** Fricke-XO-Pluronic F-127, radiotherapy dosimetry, 3D dosimeter, radiochromic dosimeter, ionizing radiation, sol-gel transition

## Abstract

Fricke-XO-Pluronic F-127 has recently been proposed as a 3D dosimeter for radiotherapy. It contains the typical ingredients of the Fricke ionizing radiation dosimeter, which are embedded in a physical gel of poly(ethylene oxide)-*block*-poly(propylene oxide)-*block*-poly(ethylene oxide) (Pluronic F-127). The main reactions upon irradiation are the conversion of Fe^+2^ to Fe^+3^ and the formation of a colored complex with XO ([XO-Fe]^+3^). The study attempts to optimize the dosimeter in terms of its solution-to-gel transition temperature. In order to lower this temperature, the use of NaCl salt has been proposed. The new composition was characterized in order to obtain information on its thermal performance, storage stability, dose response to irradiation with a medical accelerator emitting different types of radiation, and tissue equivalence. The results obtained show an improvement in the sol-gel transition temperature and dose sensitivity compared to the composition without NaCl and broaden the knowledge of the Fricke-XO-Pluronic F-127.

## 1. Introduction

The idea of embedding the well-known Fricke solution in a polymer matrix in order to obtain a 3D dosimeter for use in radiotherapy was first proposed in the early 1980s [1,2]. The spatial distribution of the dose was read using magnetic resonance imaging (MRI). At the end of the last century, there was the idea of measuring dosimeters by optical methods such as optical computed tomography. This resulted in the first Fricke 3D radiochromic dosimeter [3]. This dosimeter contained xylenol orange (XO), an organic dye that can bind ferric ions present in the irradiated dosimeter and form a colored complex. Currently, XO remains the most popular dye used in Fricke radiochromic dosimetry [4,5,6,7]; however, in recent years, methylthymol blue (MTB) has also been investigated as a potential alternative to xylenol orange [8,9].

The polymer gel matrix is a key component of 3D dosimeters. It allows for significant reduction in the diffusion of irradiation products from the irradiated to the non-irradiated area of the dosimeter. Over the years, the most popular were gel matrices made of natural and synthetic polymers, such as gelatin [10,11,12] and agarose [13,14] as natural polymers, and poly(vinyl alcohol) PVA [15,16] corresponding to synthetic polymers. Gelatin shows high transparency, much higher than agarose. Moreover, it can be dissolved at a lower temperature than agarose, which facilitates the preparation of the matrix. However, one of the most significant disadvantages of the gelatin matrix is its relatively low sol-gel transition temperature, which is about 30 °C, but in practice the stability of the gel may be affected by a temperature exceeding 23 °C [17]. Another disadvantage is the degradation of gelatin observed in some 3D polymer gel dosimeters [18]. Attempts have been made to improve the thermal stability of gelatin dosimeters, e.g., by adding formaldehyde to the MAGIC dosimeter [19]. This modification increased the solution to gel transition temperature to 69 °C [20], however, formaldehyde is a known toxin [21]. When used as a cross-linking agent, its residual concentration contributes to the toxicity of the gel dosimeter. Another attempt was to add 0.5 % *w*/*w* agarose to the gelatin matrix [22], which increased the sol-gel stability to about 60 °C, but the sensitivity of the dosimeter decreased significantly. In turn, it should be emphasized that the Fricke-based 3D gel dosimeters may be not stable at elevated temperatures. A too high storage temperature and long storage at such a temperature darkens the color, which makes optical measurements for such dosimeters impossible. Testing such dosimeters at a low temperature [4] maintains the stable color of the dosimeter for a longer time.

Recently, a new Fricke-XO dosimeter with a matrix of poly(ethylene oxide)-*block*-poly(propylene oxide)-*block*-poly(ethylene oxide) (Pluronic F-127) [23,24] has been developed and described. Pluronic F-127 forms a physical gel characterized by a high degree of transparency and colorlessness. Moreover, it is a non-toxic material (approved by the Food and Drug Administration, FDA, USA) [25,26]. Pluronic F-127 is a gel in a wide temperature range; it remains a physical gel from 20–80 °C at 25 % w/w copolymer concentration [27]. The sol-gel transition temperature may be too high during the storage or transport of dosimeters when the ambient temperature drops below 20 °C. However, it has been shown that the addition of sodium chloride can lower the sol-gel transition temperature of Pluronic F-127 [27]. This was also confirmed for another 3D polymer gel dosimeter, PAGAT2-Pluronic F-127 [28]. In this dosimeter, 1 M NaCl was used, which significantly lowered the solution to gel transition temperature below 5 °C. A preliminary follow-up study (unpublished) showed that a much lower concentration of NaCl (0.2 M) may be sufficient to adequately improve the stability of Pluronic F-127-based gel dosimeters below 20 °C. This NaCl concentration was used in the present study. In turn, the complex reaction mechanism of the Fricke dosimeter for different types of radiation, including radiation yields of Fe^+3^ formation, impact of impurities and additives, as well as irradiation temperature on the transformation of Fe^+2^ into Fe^+3^, were thoroughly discussed elsewhere [29,30,31,32].

This study was designed to optimize the Fricke-XO-Pluronic F-127 with respect to the sol-gel transition temperature. The intention was to lower this temperature so that the gel-sol conversion process would start well below 20 °C. In general, 3D dosimeters based on Pluronic F-127 (composed of 25% Pluronic F-127) are stable at elevated temperatures, with no firm dissolution observed at temperatures up to 80 °C. Lowering the storage temperature below 20 °C converts the physical Pluronic F-127 gel to sol, resulting in complete liquefaction at just a few degrees below 20 °C. Therefore, it was important to study the improvement in the thermal performance of Fricke-XO-Pluronic F-127 below 20 °C. The following reasons may also be mentioned: (i) the dosimeter may be affected by the varying transport temperature; an unintentional drop in temperature may also affect the dosimeter and the recorded 3D dose-loss of 3D dose integrity would be likely; (ii) we are currently trying to develop Fricke-XO-Pluronic F-127 to prepare 3D lung mimicking dosimeters and combined (composite) dosimeters, meaning one vial filled with volumes of Fricke-XO-Pluronic F-127 mimicking various tissues such as lungs and muscles. To accomplish this goal, the gel-sol transition temperature of this dosimeter has to be lower. The following characteristics of the resulting dosimeter with sodium chloride were performed: (i) analyses of thermal properties using two methods: the water bath test and differential scanning calorimetry (DSC) measurements, (ii) evaluation of stability over the time of storage, (iii) dose response studies on medical accelerator irradiation emitting different types of radiation with specific energy, and (iv) tissue equivalency analysis.

## 2. Materials and Methods

### 2.1. Preparation of Dosimeter

Fricke-XO-Pluronic F-127 radiochromic dosimeter samples were prepared using 25% w/w Pluronic F-127 (Pluronic^®^ F-127, Sigma-Aldrich, BioReagent, Saint Louis, MO, USA), 50 mM sulfuric acid (H_2_SO_4_, Chempur, Piekary Śląskie, Poland), 1 mM ammonium iron (II) sulfate hexahydrate ((NH_4_)_2_Fe(SO_4_)_2_·6H_2_O, FAS, Chempur, Piekary Śląskie, Poland), 0.165 mM xylenol orange disodium salt (XO, Sigma-Aldrich, Saint Louis, MO, USA), and 0.2 M sodium chloride (NaCl, Polish Chemical Reagents, Gliwice, Poland). The NaCl concentration chosen is the result of a preliminary (not published) follow-up study [28], which showed that it should sufficiently improve the stability of the dosimeter below 20 °C. The preparation of the solution was always started by dissolving Pluronic F-127 (as a white powder) in deionized water according to the procedure described elsewhere [33].

The approach to dissolving the remaining ingredients was as follows. Sulfuric acid and FAS were added to a beaker; first sulfuric acid, then FAS, and water (~7.5 mL) was poured over these compounds followed by careful mixing. XO was added to another beaker and mixed with water (~7.5 mL). The XO solution was then added to the sulfuric acid and FAS solution and stirred. If the dosimeter composition contained NaCl, it was added to the solution of sulfuric acid, FAS, and XO. Then, the solution of sulfuric acid, FAS, and XO (~23 °C) (with NaCl if required) was added to the Pluronic F-127 solution (4 °C) and the mixture was stirred very gently (note that the Pluronic F-127 solution can be easily foamed by vigorous stirring). The solution was transferred to poly(methyl methacrylate) (PMMA) UV-Vis spectrophotometric cuvettes (1 cm optical path) and glass vials (jars; height with a cap: 60 mm, outside diameter in the middle of the jar: 38 mm, maximal volume: 40 mL), which were half filled. The samples in PMMA cuvettes were stored as follows: (i) at room temperature (~23 °C) and exposed to daylight (direct sunlight through glass window), (ii) at room temperature and not exposed to daylight, and (iii) at about 4 °C in a refrigerator and protected from light. The samples that were scheduled for irradiation were kept protected from daylight in a refrigerator at 4 °C.

### 2.2. Thermal Stability Test 1

To estimate the sol-gel transition temperature of the Fricke-XO-Pluronic F-127 dosimeter, the samples in the glass jars were used. Prior to measurements, all samples were stored at room temperature (~23 °C) until the sol-gel transition of the dosimeter occurred. Then, the jars were placed in a water bath container (Donserv, Warsaw, Poland; a thermostat Huber, Compatible Control CC1, Offenburg, Germany). The initial temperature of the water, and thus the dosimeter in a jar, was 45 °C and decreased by 5 °C after each assessment until it reached 25 °C. The dosimeter temperature was then lowered by 1 °C after each assessment to a final temperature of 15 °C. After each temperature change, the samples were thermostated for 10 minutes. This was assumed to be sufficient for the gel to reach the desired temperature in the center of the sample (but not measured) as the temperature drop was slow (~1 °C /15 min) due to the large volume of the water bath (~30 L). Afterwards, they were taken out of the water and examined by tilting the jar to see if the gel inside was melting. Note that the color of this dosimeter can be unstable at elevated temperatures, for example, the initial temperature of this experiment, which was 45 °C (the direct correlation between the color of the dosimeter and a wide range of storage temperatures has not been investigated). Therefore, all optical measurements should rather be made at room temperature or below.

### 2.3. Thermal Stability Test 2

Differential scanning calorimetry (Q200, TA Instruments, New Castle, DE, USA) was used to estimate the sol-gel transition temperature of the Fricke-XO-Pluronic F-127 (with and without 0.2 M NaCl). The instrument was calibrated for both temperature and enthalpy using indium (melting point and heat of fusion are 156.6 °C and 28.57 J g^−1^, respectively). Then, the samples of Fricke-XO-Pluronic F-127 were hermetically sealed in aluminum pans and analyzed. Measurements started from 20 °C to 0 °C. In the next step, they were heated to 40 °C and then cooled to 0 °C. For the final cycle, the samples were heated to 80 °C and cooled to 0 °C. The rate of both cooling and heating was 2 °C/min.

### 2.4. Irradiation

The samples of the Fricke-XO-Pluronic F-127 dosimeter with and without NaCl were irradiated with the following two sources of ionizing radiation: a technical accelerator (ELU 6-E Elektronika, Moscow, USSR) and a medical accelerator (TrueBeam, Varian, Palo Alto, CA, USA). In the case of the technical accelerator, the samples were irradiated in air and positioned perpendicular to the electron beam (6 MeV). The duration of a single pulse was 17 ns and the frequency was 20 Hz. The dose rate was about 0.7 Gy/s (accuracy of dose rate measurements was ±10%). Only those samples that were used for thermal stability test 1 (Section 2.2) were irradiated with the technical accelerator (they were conditioned to ~23 °C before irradiation). In the case of a medical accelerator, the samples were placed 5 cm below the water surface in a water phantom (Blue Phantom 2, IBA, Germany; temperature ~23 °C) and irradiated with a 6 MV, 10 MV, or 15 MV photon beam and a 12 MeV electron beam (dose rate: X6 0.2273 Gy/s; X10 FFF (Flattening Filter Free) 0.4102 Gy/s; X15 0.1094 Gy/s; e12 0.165 Gy/s; field size 20 × 20 cm^2^, SSD 95 cm). The samples were irradiated in the accelerator isocenter perpendicular to the beam axis. The dose values were obtained on the basis of the dosimetric measurements using the CC04 ionizing chamber and the Dose-1 electrometer (IBA Dosimetry GmbH, Schwarzenbruck, Germany).

### 2.5. UV-Vis Spectrophotometry Measurements

UV-Vis spectrophotometry was used for absorbance measurements of the Fricke-XO-Pluronic F-127. For this purpose, the Jasco V-530 instrument (Tokyo, Japan) was used, and the spectra were recorded over the wavelength range of 350–700 nm with 1 nm resolution. Each sample was measured with a reference to air, since the PMMA of the cuvette contributed marginally to the UV-Vis spectrum of the dosimeter (the mean absorbance of the PMMA cuvette used is equal to 0.068 ± 0.003 for the wavelength range 350–700 nm). Thus, whenever an absorbance or optical density (Δµ) is discussed, it is understood as the absorbance or optical density of a Fricke-XO-Pluronic F-127 with a PMMA cuvette. The optical density was calculated as follows: Δµ = (ln(10)/x) × (AI – AU), where x = 1 cm is the length of the light path through the vial, AI is the measured absorbance of the irradiated sample, and AU is the absorbance of the non-irradiated sample; in both cases, the absorbance values at 585 nm were used for the calculations. The calculation of optical density was analogous to that used elsewhere [24] to ease comparisons with other radiochromic dosimeters.

### 2.6. Tissue Equivalence

The density (ρ) of the Fricke-XO-Pluronic F-127 radiochromic gel dosimeter and the elemental composition (% by weight) were obtained. The density was measured in this study at a room temperature of about 23 °C by weighting seven 10 mL volumetric glass flasks filled with the dosimeter to the volume indicated on the flasks by the manufacturer (accuracy ±0.1 mL, ±0.1 mg). It should be noted that the Pluronic F-127-based dosimeter may expand upon gelling in contrast to the gelatine-based dosimeters, which may shrink upon gelling. This should be taken into account when filling volumetric flasks with dosimetric solution. Additionally, a part of the dosimeter solution may remain on the neck of the volumetric flasks. In this case, the dosimeter in the volumetric flask was placed in a refrigerator at 4 °C so that the remnants of the dosimeter would become a solution and flow down the flask. Once the dosimeters were prepared in the volumetric flasks, they were stored for about 10 min to equilibrate to room temperature (23 °C) and weighted.

The following calculations were also performed: effective atomic number (Z_eff_), atomic-to-mass number ratio (<Z/A>), and electron density (ρ <Z/A>), based on an approach reported elsewhere [34]. The mass attenuation coefficients as a function of radiation energy for Fricke-XO-Pluronic F-127 with and without 0.2 M NaCl and water were calculated using the XCOM Program of the National Institute of Standard and Technology (NIST, Gaithersburg, MA, USA).

## 3. Results and Discussion

### 3.1. Impact of NaCl on Thermal Stability

**Figure 1:**
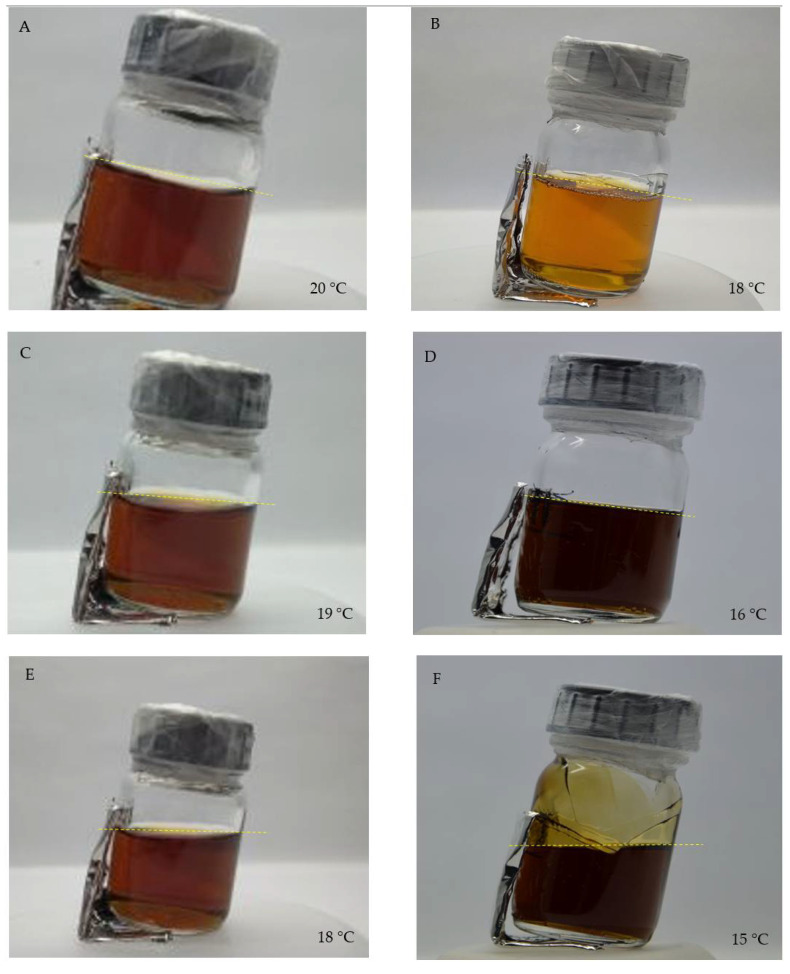
Photographs of non-irradiated Fricke-XO-Pluronic F-127 without (**A**,**C**,**E**) and with 0.2 M NaCl (**B**,**D**,**F**) during Test 1 observations. The temperatures of the gels are indicated in the photographs. The colour change of the dosimeter occurred after 5 days of experiment. The initial colour of the dosimeter is light yellow-brown as observed in (**B**). The yellow dashed lines are to indicate the surface of the dosimeter at different temperatures.

The Test 1 assessment of the sol-gel transition temperature of the non-irradiated Fricke-XO-Pluronic F-127 with and without NaCl is presented in Figure 1. The transition occurs at different temperatures related to the presence of NaCl. At a temperature of 18–19 °C, a gel-sol transition is initiated for the dosimeter without NaCl. During storage or transportation, the dosimeter may be unintentionally exposed to such temperatures that would cause it to melt. Therefore, it is reasonable to lower the gel-sol transition temperature. It was observed that for the composition of the dosimeter enriched with NaCl, there was a decrease in the temperature of the gel-sol transition. It was estimated to be in the range of 16–15 °C for 0.2 M NaCl in the dosimeter. In Figure 1, different colors of the samples can be noticed. The colour change in the dosimeter samples is related to the self-oxidation of Fe^+2^ and the formation of a complex with XO ([XO-Fe]^+3^) during the experiment.

**Figure 2:**
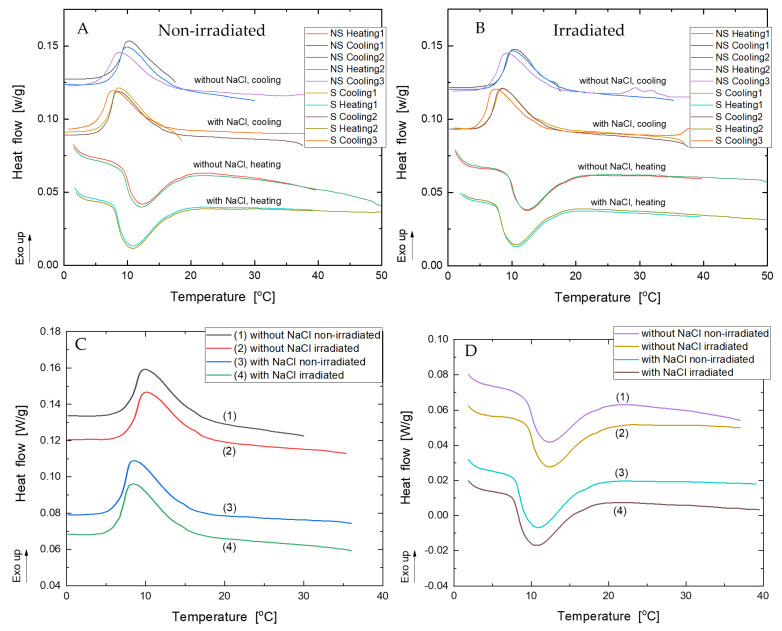
DSC results for irradiated (30 Gy) and non-irradiated Fricke-XO-Pluronic F-127 with and without 0.2 M NaCl. DSC measurements were carried out for both heating and cooling of the samples. (**A**) is for 1–3 heating and cooling cycles for non-irradiated samples, (**B**) is for 1–3 heating and cooling cycles for irradiated samples, (**C**) is for second cooling for irradiated and non-irradiated samples with without NaCl, and (**D**) is analogous to (**C**), except for the first heating cycle (NS denotes no NaCl, S denotes that the dosimeter contains NaCl).

Following Test 1, the DSC measurements were employed for detail analysis of the sol-gel-sol transition of Fricke-XO-Pluronic F-127 with and without NaCl. In this case, both irradiated (30 Gy) and non-irradiated samples were investigated. The irradiated sample was measured because it was observed for Pluronic F-127 gel (without other ingredients) that irradiation can influence the sol-gel transition temperature [28]. The dose was chosen to be higher, but close to the maximum of the dosimeter linear dose response based on the previous study [24]. The results are shown in Figure 2 and Table 1. The results in Figure 2 demonstrate the transition of the dosimeter upon heating and cooling, where the entire process is visible as follows: the temperature of the initiation of the process, the temperature for the maximum heat of the process, and the temperature at which the process finalizes. From the point of view of application, both sol-gel (at heating) and gel-sol (at cooling) transitions are important. These may not be transitions at exactly the same temperatures. For instance, the temperature at which the maximum thermal effect occurs may not be the same at heating and cooling. This can be clearly observed for the Fricke-XO-Pluronic F-127 results in Figure 1. It is also the case for all samples tested that the temperature at which the maximum thermal effect occurs upon heating is about 2 °C higher than that for cooling the dosimeter samples (T_h_ versus T_c_ in Table 1). This means that when the dosimeter is prepared and stored at, e.g., room temperature, its dissolution–conversion from gel to sol is possible at lower temperatures compared to the formation of gel from a dosimetric solution. Thus, the dosimeter is more resistant to unfavorable dissolution and loss of 3D integrity in the event of unintentional cooling during storage or transport. It is also characteristic that the first and second cooling/heating cycles produced similar parameters of transitions temperatures (Table 1). However, the third cooling of the Fricke-XO-Pluronic F-127 decreased the transitions temperatures. This denotes that the third-time cooled dosimeter is more resistant to transition into a solution, which seems favorable regarding the unintentional cooling of the dosimeter during storage or transportation. Such results suggest that the temperature history of the dosimeter affects its thermal properties, and that subjecting the dosimeter to heating and cooling cycles may lower the lower gel-sol transition temperature, thereby increasing the stability of the system. It should also be reported that no turbidity/additional increased scattering of light was observed (naked eye observations) for repeated melting and gelation (2–3 times) of pure 25% Pluronic F-127 solution; the solution/gel remained crystal clear. However, this issue was not investigated in detail. Additionally, it is clear from Figure 2 and Table 1 that this study does not provide results showing a clear effect of irradiation on the Fricke-XO-Pluronic F-127 transition temperatures, contrary to the observations for 23% Pluronic F-127 (without additional ingredients) reported elsewhere [28].

### 3.2. Temporal Stability of Fricke-XO-Pluronic F-127

**Figure 3:**
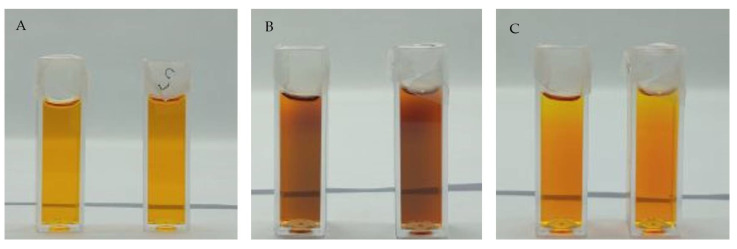
Photographs of the Fricke-XO-Pluronic F-127 dosimeter samples without and with 0.2 M NaCl, which were stored at room temperature and exposed to daylight: (**A**)—immediately after preparation, (**B**)—24 h, (**C**)—48 h. The samples on the right-hand side contain NaCl.

**Figure 4:**
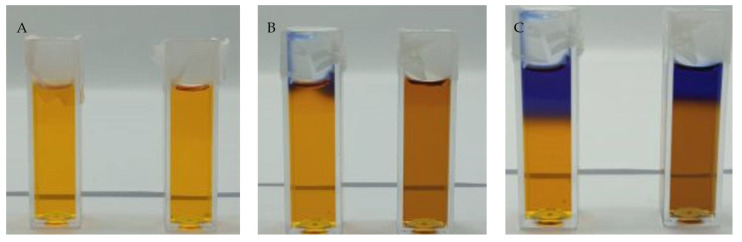
Photographs of the Fricke-XO-Pluronic F-127 dosimeter samples without and with 0.2 M NaCl, which were stored at room temperature and not exposed to daylight: (**A**)—immediately after preparation, (**B**)—24 h, C – 48 h. The samples on the right-hand side contain NaCl.

**Figure 5:**
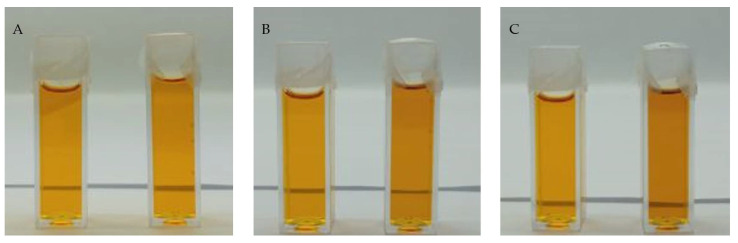
Photographs of the Fricke-XO-Pluronic F-127 dosimeter samples without and with 0.2 M NaCl, which were stored at about 4 °C in a refrigerator and protected from light: (**A**)—immediately after preparation, (**B**)—24 h, (**C**)—48 h. The samples on the right-hand side contain NaCl.

The temporal stability of Fricke-XO-Pluronic F-127 without and with 0.2 M NaCl was tested under the following three different storage conditions: (i) at room temperature and exposed to daylight (direct sunlight through glass window) (Figure 3), (ii) at room temperature and not exposed to daylight (Figure 4), and (iii) kept at about 4 °C in a refrigerator and protected from light (Figure 5). Observations with the naked eye during the storage of dosimeter samples lead to the following conclusions: (i) the dosimeter is unstable after preparation regardless of storage conditions and the presence of NaCl; (ii) the presence of NaCl deteriorates the stability of the dosimeter; (iii) after preparation, the dosimeter with and without NaCl should be kept at low temperature and protected from light; (iv) if irradiated, it should be stored at a low temperature (e.g. 4 °C) and be protected from light or protected from light at room temperature; it should be noted that in low temperatures the dosimeter may liquefy and thus may lose the recorded dose distribution; (v) the highest stability of samples was observed for samples stored at 4 °C protected from light; those stored at room temperature, protected from light or not, can be stored for no longer than 24 h; at this time, the dosimeter can be used somehow for dosimetry, however, the golden rule for this dosimeter is to consume it for dosimetric purposes within a couple of hours after taking it out of the refrigerator; and (vi) it is clear that the dosimeter composition is subject to complex transformations over storage, which are related to the presence of NaCl, the impact of temperature and light, and the period of storage; these are indicated by complex colour transformations from pale yellow to brown, dark blue, turquoise, dark green, and lemon yellow (last three now shown in this work). Certainly, these colors correspond to the different chemical structures developing in Fricke-XO-Pluronic F-127; however, they were not investigated in this work.

**Figure 6:**
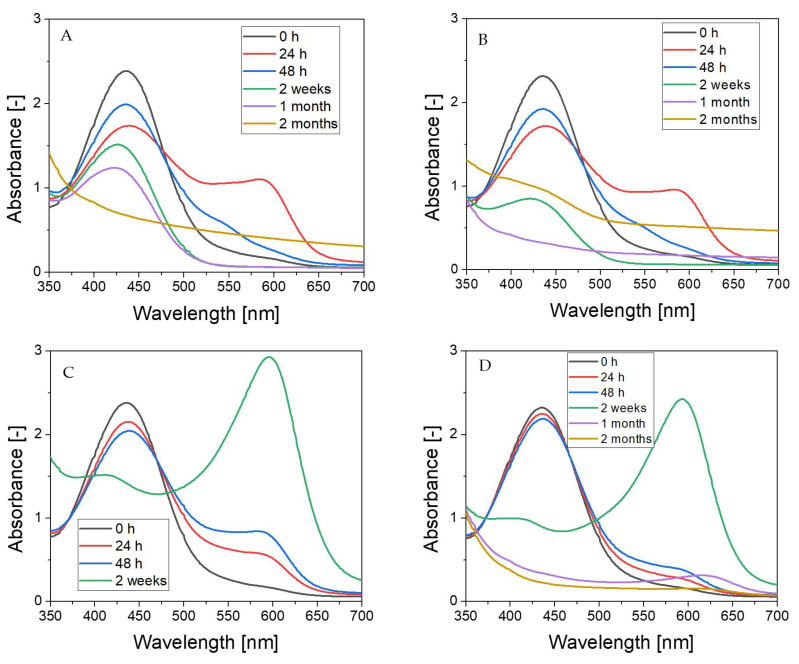

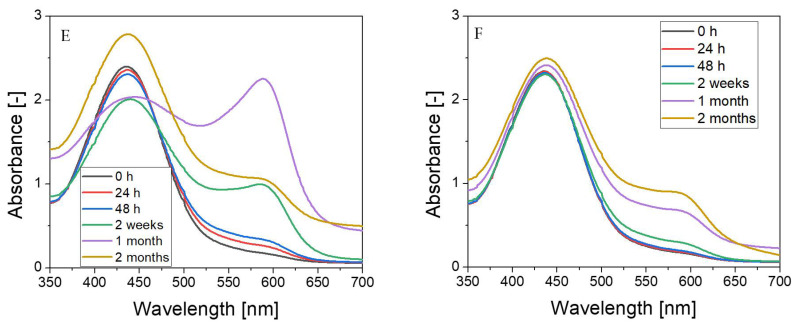
Absorbance spectra of the Fricke-XO-Pluronic F-127 dosimeter without and with 0.2 M NaCl stored under different conditions: (**A**)—with NaCl, stored at room temperature, and exposed to light; (**B**)—without NaCl, stored at room temperature, and exposed to light; (**C**)—with NaCl, stored at room temperature, protected from light; (**D**)—without NaCl, stored at room temperature, protected from light; (**E**)—with NaCl, stored at about 4 °C in a refrigerator, and protected from light; and (**F**)—without NaCl, stored at about 4 °C in a refrigerator, and protected from light.

The Fricke-XO-Pluronic F-127 without and with 0.2 M NaCl was analyzed by using UV-Vis spectrophotometry over the time of storage under different conditions as reported above. The results are shown in Figure 6. It should be noted that Figure 6A and B correspond to Figure 3, Figure 6C and D correspond to Figure 4, and Figure 6E and F correspond to Figure 5. In general, the initial spectrum that was recorded after the preparation of the dosimeter with or without NaCl evolves over storage. Two maxima corresponding to bands at around 435 and 585 nm should be considered. Their evolution is related to the formation and disappearance (transformation) of the ([XO-Fe]^+3^) complex. These, for Fricke-XO-Pluronic F-127 with and without NaCl, stored at room temperature and exposed to light, transform according to a similar pattern (Figure 6A,B). During the first 24 h, the maximum at about 435 nm decreases and the one at about 585 nm builds up—the samples darken towards brownish (Figure 3B). However, during the next 24 h, there is an opposite trend; the maximum at 435 nm increases and the one at 585 nm decreases—the samples bleach (Figure 3C). Upon bleaching, at up to 1 month, the band at about 435 nm shifts towards the shorter wavelengths; however, it only disappears for the sample without NaCl. Storage of over 1 month produces spectra without specific bands—the absorbance increases in the examined wavelength range (Figure 6A,B); the samples change color to lemon yellow. The absorbance spectra of samples stored under similar conditions, but protected from light, change over time in a similar manner only during the first 24 h (Figure 6C,D). The next 24 h of storage is related to a further increase in the intensity of the 585 nm band, which was not the case for the samples exposed to light; the samples become darker brown (Figure 4C). Two weeks of storage produces a high intensity band shifted towards higher wavelengths. This corresponds to a transformation of color from brownish to dark blue. With a longer storage time, the band decreases significantly and shifts above 600 nm. This causes the samples to turn green (not shown in Figure 4). Note that the sample with NaCl could not be measured due to significant evaporation (the samples were covered with one layer of Parafilm^®^). This sample turned yellow after 2 months of storage. 

The most stable were the samples that were stored in a refrigerator at 4 °C, protected from light (Figure 6E,F). The presence of NaCl sensitized the dosimeter to a color change, analogous to the samples stored under all tested conditions. Additionally, the color of the samples converted from yellowish brown to dark brown; no other colors were observed during long storage (see also Figure 5). The sample containing NaCl bleached after 2 months of storage (Figure 6E), although it still remained dark brown.

### 3.3. Dose Response of Fricke-XO-Pluronic F-127 with NaCl 

**Figure 7:**
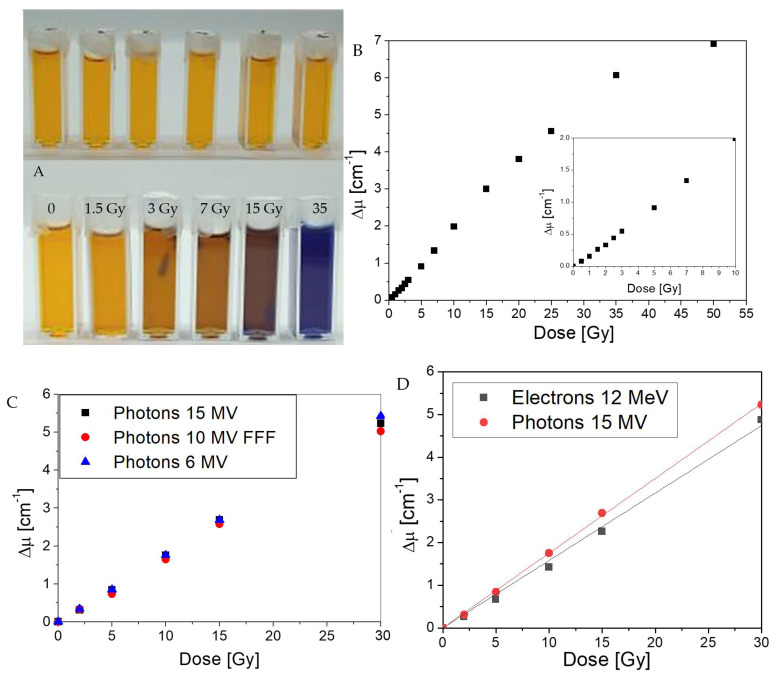
Dose response of Fricke-XO-Pluronic F-127 containing 0.2 M NaCl: (**A**)—photographs of dosimeter samples before and after irradiation; (**B**)—optical density as a function of radiation dose for the dosimeter; measurements performed about 1 h after irradiation; inset: same relation but for a narrow dose range of 0–10 Gy; irradiation was performed with 6 MV photons; (**C**)—dose response of the dosimeter for samples irradiated with 6 MV, 10 MV FFF, and 15 MV photons; and (**D**)—dose response of the dosimeter for samples irradiated with 15 MV photons and 12 MeV electrons.

The ionizing radiation dose response of Fricke-XO-Pluronic F-127 with 0.2 M NaCl was examined after irradiation with a medical accelerator. The results are shown in Figure 7. The immediate effect of the irradiation is a color change (Figure 7A), analogous to the observations presented in the previous chapters. The yellow color samples convert to darker brown and then to dark blue at over 30 Gy. The calculated optical density correlated with the radiation dose (Figure 7B) revealed that the dynamic dose range is around 55 Gy, and the dose threshold is lower than 0.5 Gy (0.5 Gy was the lowest dose applied, and a clear change in optical density was observed for this dose). The dosimeter responded to 6 MV, 10 MV FFF and 15 MV photons in a similar manner (Figure 7C); however, the response to irradiation with 12 MeV electrons was slightly lower than that of photons (Figure 7D).

Following the optical density results presented in Figure 7, the dose sensitivity of Fricke-XO-Pluronic F-127 with 0.2 M NaCl was investigated for different irradiations and dose ranges (Table 2). The dose sensitivity for irradiation with electrons evidently deviated from that for photons. Thus, it is clear that Fricke-XO-Pluronic F-127 should be calibrated separately for electrons and photons, if different applications in radiotherapy dosimetry are required. The dose sensitivity is related to the dose range used in its calculation (Table 2); it appears to be decreasing for the higher dose ranges above 20 Gy, despite the high R^2^ coefficient indicating good linearity. It also follows from the calibration containing the highest number of points (Figure 7B) that the linear dose range is not greater than 25 Gy.

In summary, there are similarities and differences between Fricke-XO-Pluronic F-127 with and without [24] NaCl, as follows: (i) both have similar dynamic and liner dose ranges, and a low dose threshold (lower than 0.5 Gy; so far not established); (ii) the composition with NaCl has greater dose sensitivity; without NaCl it is about 0.1316 ± 0.0022 Gy^−1^ cm^−1^; (iii) both respond similarly to irradiation with 6 and 15 MV photons (the composition without NaCl was not tested for dose response with 10 MV FFF photons and electrons); (iv) the addition of NaCl deteriorates the dosimeter’s storage stability; and (v) the conclusion of this and the former study [24] is that the dosimeter with and without NaCl should be kept at a low temperature (e.g., 4 °C) in the dark; eventually, in the dark at room temperature after irradiation.

The comparison of Fricke-XO-Pluronic F-127 (with or without NaCl) with other Fricke gel dosimeters based on gelatine, agarose, or poly(vinyl alcohol) (PVA) matrices [35,36] leads to the following conclusions: (i) Fricke-XO-Pluronic F127 can be used for optical measurements, and therefore probably for 3D optical computed tomography (so far not studied), which is similar to Fricke gels with gelatine, agarose, and PVA matrices; note that PVA can also form an opaque cryogel, in addition to a transparent hydrogel, that prevents optical measurements, but MRI can still be used [35]; (ii) Fricke-XO-Pluronic F127 was not examined with NMR/MRI readout, unlike the other dosimeters mentioned; thus, we can only assume such a possibility; however, it is know that Fricke-XO-Pluronic F127 suffers from Fe^+3^ diffusion [23] similar to the other dosimeters (see Table 2 in [23]), which would probably limit its use with MRI readout; it requires further optimization; (iii) lowering the sol-gel transition temperature of Fricke-XO-Pluronic F-127 by adding NaCl also allows this dosimeter to be measured at a lower temperature than room temperature, similar to the dosimeters with the other matrices; it can also extend the time period available for 3D measurements related to autooxidation (it is slower below room temperature); and (iv) NaCl played a double role in Fricke-XO-Pluronic F-127: it improved the sol-gel transition temperature and increased the dose sensitivity with respect to the dosimeter without NaCl. The chemical reactions in the Fricke solution and gel dosimeters, including the effect of NaCl on Fe^+3^ formation, were discussed earlier [29,30,31,32,35,36]; some authors found no significant impact of NaCl on FXG (Fricke Xylenol Gel) dosimeters [37].

### 3.4. Tissue Equivalence of Fricke-XO-Pluronic F-127 Dosimeters

**Figure 8:**
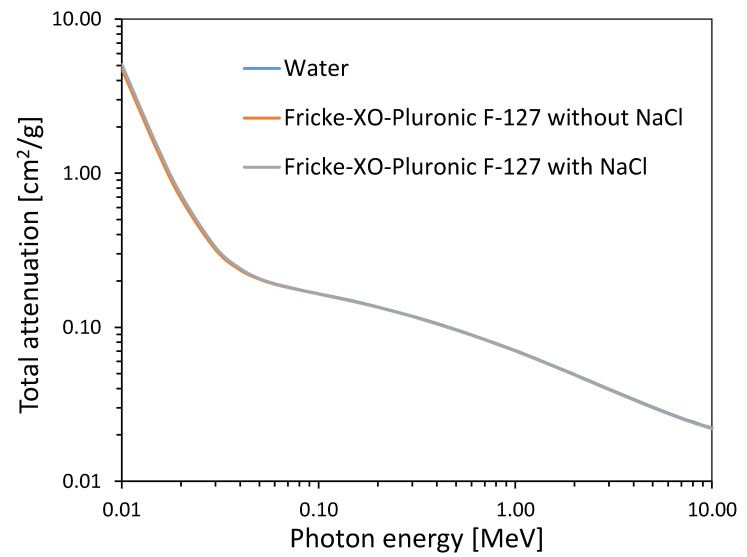
Total attenuation coefficient with coherent scattering as a function of the photon energy for water and Fricke-XO-Pluronic F-127 without and with 0.2 M NaCl.

The water equivalence, expressed as effective atomic number (Z_eff_), elemental composition, physical density (ρ), atomic-to-mass number ratio (<Z/A>), and electron density (ρ <Z/A>) of water and the Fricke-XO-Pluronic F-127 radiochromic dosimeter without and with 0.2 M NaCl were calculated as described elsewhere [34]. These data are presented in Table 3. Additionally, the total attenuation coefficient with coherent scattering as a function of the photon energy for water and Fricke-XO-Pluronic F-127 without and with 0.2 M NaCl is shown in Figure 8. It is clear that Fricke-XO-Pluronic F-127 dosimeters are close to water and thus are soft tissue equivalent in terms of interaction with ionizing radiation.

## 4. Conclusions

The results of the research showed the possibility of lowering the sol-gel-sol transition temperatures of the Fricke-XO-Pluronic F-127 dosimeter by adding 0.2 M sodium chloride. The transition temperature of the dosimeter is approximately 2 °C lower than that for the original dosimeter without NaCl both during heating and cooling. Thus, the goal of improving the dosimeter stability at lower temperatures has been achieved. Moreover, lowering the sol-gel transition temperature is possible by cyclically heating and cooling the dosimeter in the temperature range enabling the phase transition. It is likely that the multiple heating and cooling process can be used as one of the steps in the production of the dosimeter. However, this requires further research.

The addition of sodium chloride also allows for significant improvement in the dosimeter’s dose sensitivity to ionizing radiation in relation to the composition without NaCl. The linear and dynamic dose response remains similar to that of the NaCl-free dosimeter. The dosimeter responds to photons (6 MV, 10 MV FFF and 15 MV) in a similar manner; however, it responds slightly differently to irradiation with electrons. The dosimeter is tissue equivalent.

On the other hand, the presence of NaCl in the dosimeter’s composition negatively affects its chemical stability during storage; it is lower than that for the dosimeter without it. The irradiated dosimeter must be stored at a temperature keeping it in the form of a gel, which reduces its stability and the time allowed for measurements. Therefore, further research should focus on improving the chemical stability of the Fricke-XO-Pluronic F-127 dosimeter containing NaCl.

## Figures and Tables

**Table 1:** DSC measurement results of the irradiated (30 Gy, 6 MeV electrons) and non-irradiated Fricke-XO-Pluronic F-127 with and without NaCl for heating and cooling. T_c_ or T_h_ is the temperature at which the maximum thermal effect occurs, ΔH denotes the enthalpy change, c is for cooling, on is for onset (initial phase of a phase transition process), and h is for heating.

	T_c_ [°C]	T_c on_ [°C]	ΔH_c_ [J/g]	T_h_ [°C]	ΔH_h_ [J/g]
Non-irradiated sample without NaCl					
1 cooling/heating	10.2	16.1	4.4	12.3	4.9
2 cooling/heating	10.0	16.1	4.4	12.1	5.2
3 cooling	8.8	15.2	4.3	-	-
Irradiated sample without NaCl					
1 cooling/heating	10.3	15.0	4.4	12.3	4.9
2 cooling/heating	10.2	16.6	4.4	12.2	4.9
3 cooling	9.3	15.8	4.3	-	-
Non-irradiated sample with 0.2 M NaCl					
1 cooling/heating	8.7	15.7	5.9	10.9	4.1
2 cooling/heating	8.6	15.1	5.0	10.8	5.3
3 cooling	7.9	14.3	4.5	-	-
Irradiated sample with 0.2 M NaCl					
1 cooling/heating	8.5	14.7	4.5	10.6	5.1
2 cooling/heating	8.4	14.8	4.6	10.6	5.1
3 cooling	7.4	13.9	4.8	-	-

**Table 2:** Basic dose response features of Fricke-XO-Pluronic F-127 containing 0.2 M NaCl. Dynamic dose response is up to 50 Gy and dose threshold is lower than 0.5 Gy; 0.5 Gy was the lowest dose applied in this study. Dose sensitivity is understood to be the slope of the linear regression. Dose sensitivity ** and Intercept **are calculated from Absorbance (585 nm) vs. dose relations (not shown in this work) for 1 cm pathlength. The Dose sensitivity ** multiplied by the factor of 2.303 gives the optical attenuation coefficient according to the approach in [35].

Type of Radiation	Dose Range Examined [Gy]	Dose Sensitivity [Gy^−1^·cm^−1^]	R^2^	Dose Sensitivity ** [Gy^−1^·cm^−1^]	Intercept **	R^2^
Electrons 12 MeV (Figure 7D)	0–30	0.1580 ± 0.0035	0.997	0.0701 ± 0.0017	0.222 ± 0.025	0.997
Photons 6 MV (Figure 7B) Photons 6 MV (Figure 7C)	0–10 0–20 0–25 0–30	0.1919 ± 0.0029 0.1932 ± 0.0018 0.1885 ± 0.0020 0.1797 ± 0.0009	0.997 0.999 0.998 0.999	0.0861 ± 0.0013 0.0844 ± 0.0099 0.0813 ± 0.0012 0.0787 ± 0.0004	0.259 ± 0.006 0.265 ± 0.008 0.277 ± 0.013 0.275 ± 0.006	0.998 0.998 0.997 0.999
Photons 10 MV FFF (Figure 7C)	0–30	0.1670 ± 0.0016	0.999	0.0733 ± 0.0010	0.275 ± 0.015	0.999
Photons 15 MV (Figure 7C)	0–30	0.1751 ± 0.0011	0.999	0.0763 ± 0.0007	0.275 ± 0.011	0.999

**Table 3:** Elemental composition and tissue equivalence of Fricke-XO-Pluronic F-127 without and with 0.2 M NaCl in reference to water.

	Elemental Composition [% by weight]								ρ [g/cm^3^]	<Z/A>	ρ × <Z/A>	Z_eff_
	_6_C	_1_H	_16_O	_17_Cl	_11_Na	_16_S	_7_N	_26_Fe				
Fricke-XO-Pluronic F-127	14.23	10.72	74.80	-	5.63 × 10^−3^	0.17	0.02	0.04	1.040 ± 0.002	0.553	0.575	7.41
Fricke-XO-Pluronic F-127 + 0.2 NaCl	14.23	10.63	74.04	0.52	0.34	0.17	0.02	0.04	1.042 ± 0.003	0.552	0.575	7.58
Water	-	11.19	88.81	-	-	-			1.000	0.555	0.555	7.51

## Data Availability

The data supporting reported results is not stored in any publicly archived datasets. The readers can contact the corresponding author for any further clarification of the results obtained.
